# Regional paleofire regimes affected by non-uniform climate, vegetation and human drivers

**DOI:** 10.1038/srep13356

**Published:** 2015-09-02

**Authors:** Olivier Blarquez, Adam A. Ali, Martin P. Girardin, Pierre Grondin, Bianca Fréchette, Yves Bergeron, Christelle Hély

**Affiliations:** 1 Département de Géographie, Université de Montréal, Montréal, Québec, Canada; 2 Institut des Sciences de l’Evolution de Montpellier, CNRS—IRD—Université Montpellier 2—EPHE, Montpellier, France; 3 Natural Resources Canada, Canadian Forest Service, Laurentian Forestry Centre, Quebec, Québec, Canada; 4 Ministère des Forêts, de la Faune et des Parcs, Direction de la recherche forestière, Québec, Canada; 5 Centre de recherche en géochimie et géodynamique, Université du Québec à Montréal, Montréal, Québec, Canada; 6 Centre d’étude de la Forêt, Université du Québec à Montréal, Montréal, Québec, Québec, Canada; 7 Natural Sciences and Engineering Research Council of Canada Industrial Chair in Sustainable Forest Management, Forest Research Institute, Université du Québec en Abitibi-Témiscamingue, Rouyn-Noranda, Québec, Canada

## Abstract

Climate, vegetation and humans act on biomass burning at different spatial and temporal scales. In this study, we used a dense network of sedimentary charcoal records from eastern Canada to reconstruct regional biomass burning history over the last 7000 years at the scale of four potential vegetation types: open coniferous forest/tundra, boreal coniferous forest, boreal mixedwood forest and temperate forest. The biomass burning trajectories were compared with regional climate trends reconstructed from general circulation models, tree biomass reconstructed from pollen series, and human population densities. We found that non-uniform climate, vegetation and human drivers acted on regional biomass burning history. In the open coniferous forest/tundra and dense coniferous forest, the regional biomass burning was primarily shaped by gradual establishment of less climate-conducive burning conditions over 5000 years. In the mixed boreal forest an increasing relative proportion of flammable conifers in landscapes since 2000 BP contributed to maintaining biomass burning constant despite climatic conditions less favourable to fires. In the temperate forest, biomass burning was uncoupled with climatic conditions and the main driver was seemingly vegetation until European colonization, i.e. 300 BP. Tree biomass and thus fuel accumulation modulated fire activity, an indication that biomass burning is fuel-dependent and notably upon long-term co-dominance shifts between conifers and broadleaf trees.

Climate is a dominant driver of wildland biomass burning worldwide^1^,^2^. Amongst other things, climate acts upon biomass burning through temperature-driven evapotranspiration and drought, with both contributing to fuel build-up and fire spread^3^. Recent projections indicate that there will be an increase in fire frequency and area burned in many forest ecosystems on Earth during the coming decades in response to on-going anthropogenic climatic warming^4^,^5^, with environmental and economic consequences.

The dominant effect of climate on biomass burning varies in magnitude between regions and in many cases may be overridden by other local to regional factors such as topography, edaphic conditions, vegetation and humans^6^,^7^. Changes in vegetation types, particularly in the proportion of flammable versus non-flammable species in landscapes, impact fire ignition, propagation and recurrence^8^,^9^. Paleoecological investigations have recently highlighted the time-dependent influences of climate, vegetation and humans on biomass burning^10^,^11^. For instance, regions such as South America and Africa have recently experienced a rise in biomass burning associated with intensification of human activities like deforestation^12^. In contrast, other regions have seen a lowering of biomass burning in association with deployment of active fire suppression efforts^13^, and passive suppression consequent to the loss of forests^14^. This non-uniform contribution of different factors (climate, vegetation, humans, etc.) to biomass burning may underpin regional differences in the response of fire to future warming, and thereby pose a great challenge to the prediction of future fire activity^1^.

Sedimentary charcoal records can provide information about past changes in fire frequency, fire size, and biomass burning at different temporal and spatial scales^15^,^16^,^17^. Their careful interpretation also provides indications of the long-term factors controlling fire at these scales^18^. To date, much of our understanding of the drivers of biomass burning was gained from the analysis of ensemble-averaging of paleofire records at sub-continental to continental scales^14^. The coarse resolution of these paleofire syntheses is inherent to the low density of paleofire reconstructions in some areas^2^. This limitation poses challenges. At large scales, the merging of charcoal records from different vegetation zones, climate and human population densities, could hinder the possibility of deciphering the importance of climate versus vegetation and human controls on fire at some locations. This may be particularly true for the North American ecosystems where the population density is currently very heterogeneous and stayed low up to the colonization period in the 18th century^19^. Thus, the imprint of the temporarily and spatially-heterogeneous factors is likely being dampened by the ensemble-averaging of multiple regions, which will tend to highlight the main factors common to all regions (i.e. climate). Moreover, within this ensemble-averaging, some regions are more sampled than others. Hence, the information embedded in these records may outweigh the information contained in records from other regions where sampled sites are less numerous.

Finally, focusing on climate alone may result in an underestimation of the influence of the vegetation on the fire regime, which could be particularly important in ecosystems co-dominated by contrasted vegetation types or when changes in land use occur^20^. In the boreal forests of Alaska an increase in conifers *c.* 5500 years BP (notably *Picea mariana*) increased landscape flammability, which resulted in maintaining high fire frequency despite unfavourable climatic conditions for fire occurrence^11^. The same process occurred in the eastern North America boreal mixedwood forest where changes in the co-dominance of coniferous and deciduous taxa with different flammability characteristics likely influenced fire occurrence since at least 3000 years BP^10^. If it is now well accepted that integrating vegetation attributes into forest modelling experiments is important, particularly for the prediction of future wildfire risks^21^, conversely very few paleoecological studies provide quantified vegetation attributes to support fire histories. This lack is generally attributable to the absence of quantified sources of information regarding vegetation, and notably biomass. Such quantifications could now be facilitated with the availability of homogenized forest attribute data estimated through remote sensing and forest inventory analyses at fine resolution and over very large territories^22^.

Here we present a new spatially-explicit, regional-scale, reconstruction of the paleofire history in eastern North America covering the last 7000 years. From this reconstruction, we differentiate the role of human activities, climate and vegetation in relation to regional biomass burning at millennial time-scales. Regional biomass burning was inferred from 64 well-dated lacustrine charcoal records (Fig. 1 and Supplementary Fig. 1) and compared with (i) climate reconstructions (temperature and precipitation) issued from two general circulation models (HadCM3 and CCSM3 experiments), (ii) new quantitative reconstructions of vegetation biomass for eastern North America based on a modern analogue technique applied to pollen series and actual tree taxa biomass estimated from remote sensing analyses, and (iii) human population density extracted from the HYDE database^19^. Our analyses involved four regions based on current vegetation distribution (Fig. 1): (i) the open coniferous forest/tundra, (ii) the closed boreal coniferous forest, (iii) the boreal mixedwood forest, and (iv) the temperate forest. We hypothesize that long-term regional trends in biomass burning exist, controlled by complex and variable interactions between climate, vegetation and human land-use changes. This hypothesis is partly based on previous regional paleofire history reconstructions carried out in coniferous and boreal mixedwood forests^10^,^16^,^18^,^23^,^24^.

## Results

### Regional biomass burning history

Reconstructions of the paleofire histories across eastern North America show marked differences in trajectories during the last 7000 years (Fig. 2; 500-yr window half width smoothers). In the open coniferous forest/tundra, biomass burning increased until the mid-Holocene approximately 5000 years before present (hereafter BP), and then declined continuously until today. This decrease in biomass burning is significant, as it can be assessed by the non-overlapping 95% confidence interval between today and earlier periods. A very similar trajectory was noted in the closed boreal coniferous forest, albeit more variability prevailed prior to 5000 BP. An opposite trajectory was found in the boreal mixedwood forest reconstruction. There, biomass burning displayed an increasing trend starting ca. 4000 BP and remained high and stable over the last 2000 years. Finally, biomass burning in the temperate forest was somewhat cyclic over the period 7000 to 2000 BP with a maximum attained during the 5000–4000 BP period and a minimum around 3000–2000 BP. It is noteworthy that the biomass burning trajectory during the last 200 years or so was marked by a rapid rise to levels experienced approximately 5000 BP (Fig. 2, 200-yr window half width smoother).

**Figure 1:**
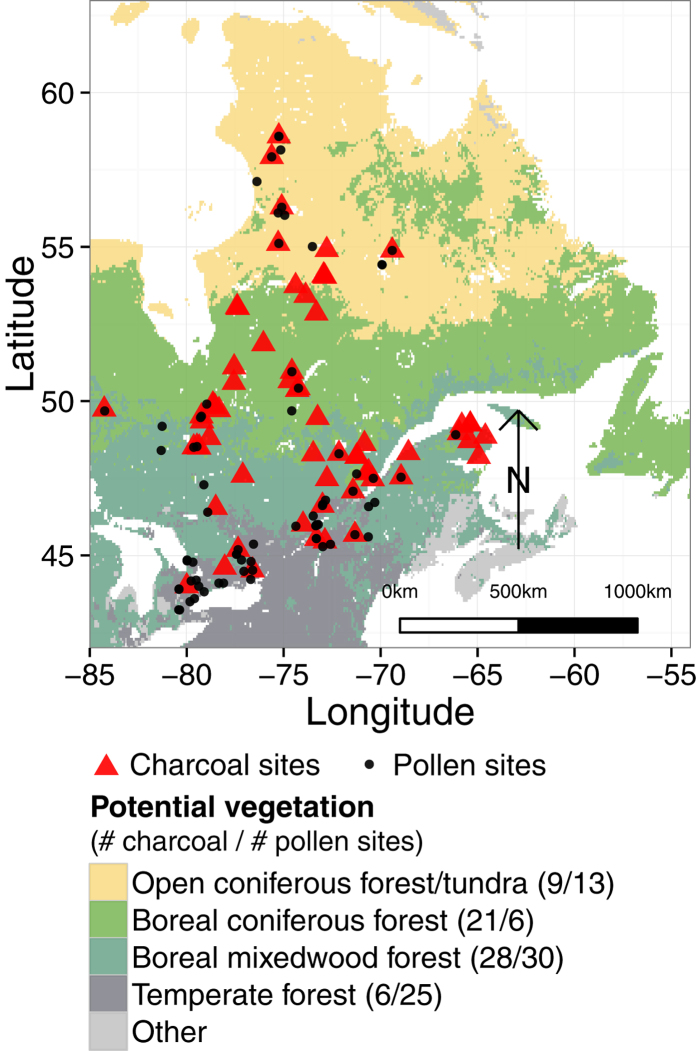
Location map of selected charcoal (red) and pollen (black) sites. Sedimentary sites are represented using different shapes according to their localization within the four vegetation zones defined for this study adapted from Ramankutty & Foley. The boreal mixedwood forest zone encompass the boreal mixedwood forest in the north and evergreen deciduous mixed forest in the south^51^.

**Figure 2:**
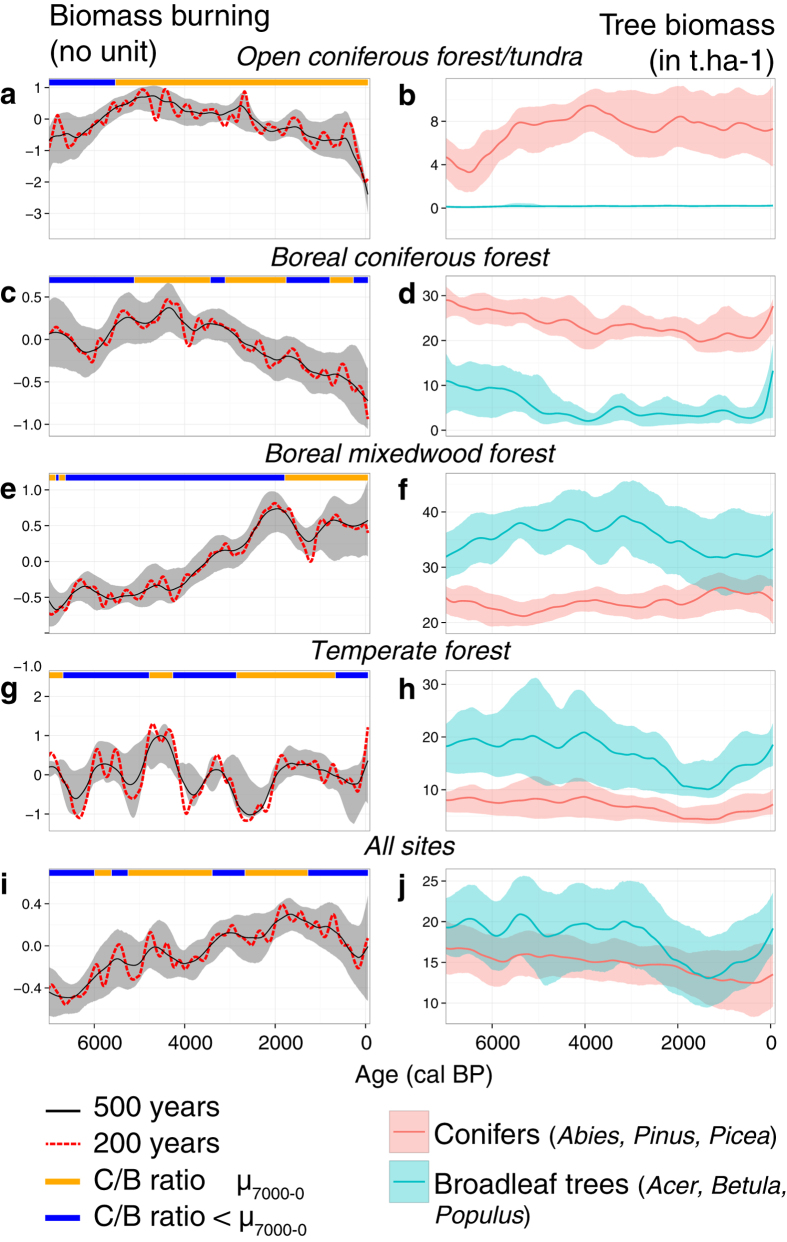
Biomass burning activity from the compositing of charcoal records and vegetation biomass expressed as total conifers and broadleaf tree biomass computed for the main tree genus, i.e. *Abies, Acer, Betula, Picea, Pinus, Populus* for the open coniferous forest/tundra (**a**,**b**), boreal coniferous forest (**c**,**d**), boreal mixedwood forest (**e**,**f**), temperate forest (**g**,**h**) and all sites (subcontinental average: **i**,**j**). The black and red lines correspond to the scatter plot smoother calculated using a 500 and 200 year window half width, respectively. The grey (biomass burning) and coloured (biomass) areas represent the 95% confidence interval calculated using the bootstrap procedure (calculated on the 500-yr trend). Blue and orange lines highlight periods when the conifer *vs* broadleaf trees ratio is above (orange) or below (blue) the long-term (7000 yr) average (see Supplementary Fig. 3).

Figure 2 also displays the paleofire history pooled at the subcontinental-scale (i.e. ‘All sites’ reconstruction). One may note that it is very similar to that of the boreal mixedwood forest’s reconstruction and this is inherent to the weighting toward regions that encompass the majority of paleoecological sites (Fig. 1). The gradual increase in biomass burning noted in this subcontinental-scale averaging is partly the result of variability occurring in the boreal mixedwood forest, and does not necessarily reflect the open coniferous forest/tundra, closed boreal coniferous forest, or temperate forest biomass burning patterns.

### Correlation analyses and linkages to processes

**Figure 3:**
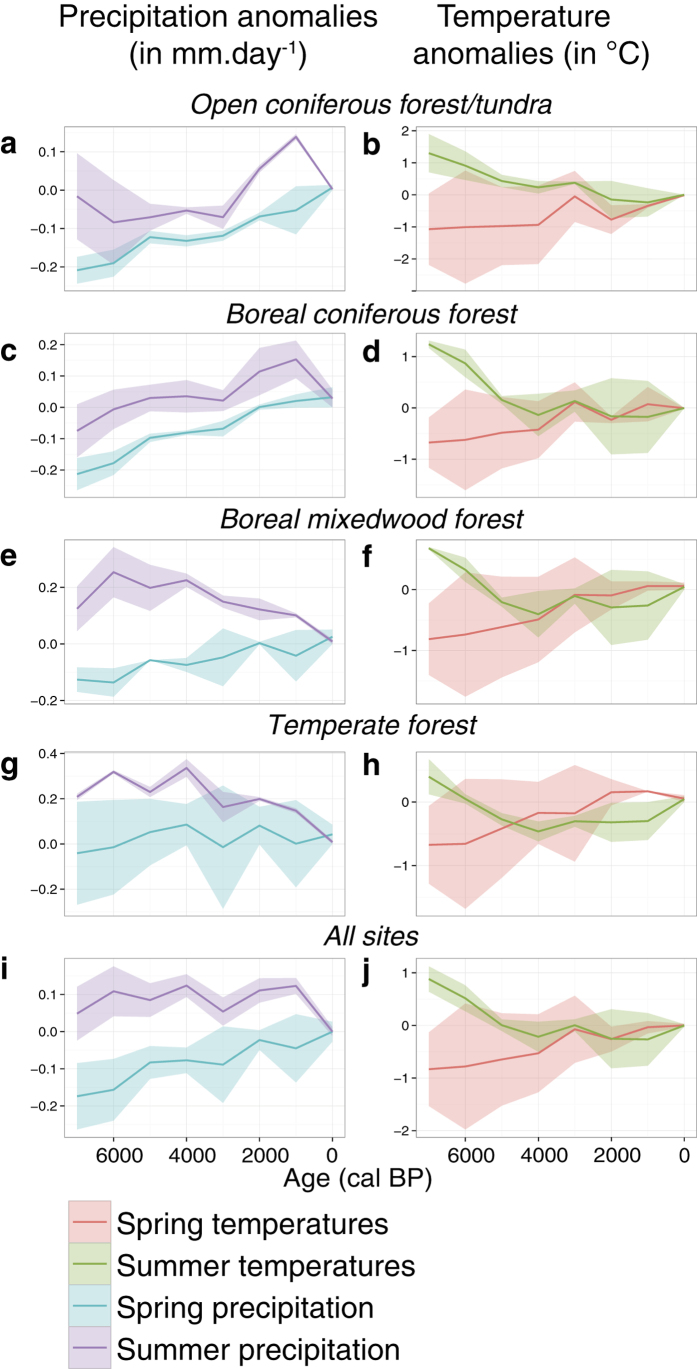
GCM spring and summer precipitation anomalies (**a**,**c**,**e**,**g**,**i** in mm.day-1) and temperature anomalies (**b**,**d**,**f**,**h**,**j** in °C) for the open coniferous forest/tundra (**a**,**b**), boreal coniferous forest (**c**,**d**), boreal mixedwood forest (**e**,**f**), temperate forest (**g**,**h**) and all sites from eastern Canada (**i**,**j**), from GCM simulations. Plain lines correspond to the average of the HadCM3 and CCSM3 models outputs, the shaded areas correspond to the range of anomalies from the two GCMs.

**Figure 4:**
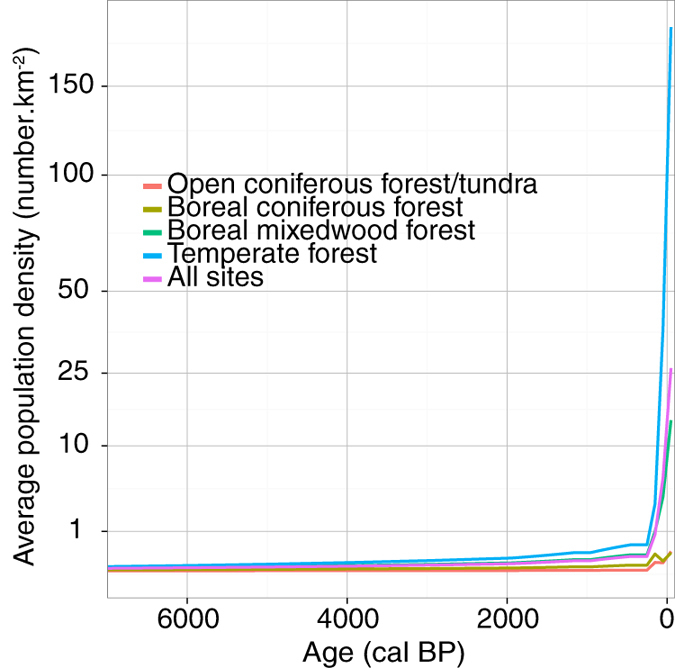
Average population density in a 100 km radius around charcoal sites for the four regions expressed as inhabitant number per square kilometre.

**Figure 5:**
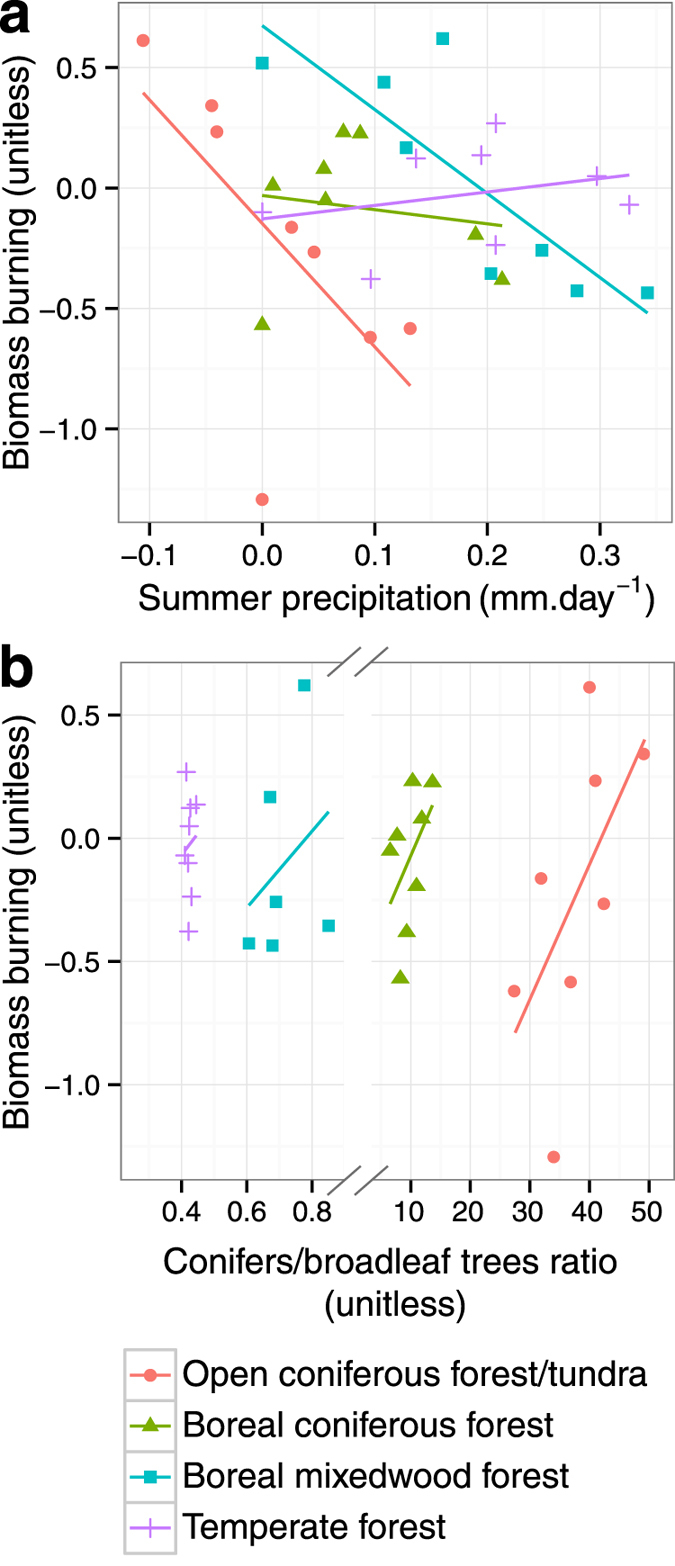
Partial least squares regression results. Regressions were performed at the region level between 1000-yr averages of biomass burning and summer precipitation anomalies (**a**) and the conifer *vs* broadleaf trees ratio (**b**).

Table 1 illustrates the semi-partial Spearman rank correlations of the regional biomass burning history with the vegetation, climate, and human population density features on millennial time-scales (illustrated in Figs 2, 3 and 4). Two conditional situations leading to high biomass burning across eastern North America are illustrated by these analyses. First, the results show that summer precipitation was negatively correlated with multi-millennial variability of biomass burning. This observation is valid independently of the use of the HadCM3 or CCSM3 climate model simulation runs, an indication that results are robust. Periods of sustained low summer precipitation favour fire-conducive days that translate into fire propagation, as suggested by analyses of modern fire statistics and fire-weather variables^25^. The relationship is particularly evident in the open coniferous forest/tundra and boreal mixedwood forest (Fig. 5), for which increases and decreases in precipitation are simulated since 7000 BP, respectively (Fig. 3). The functional form of the relationship is consistent with the decreases and increases in biomass burning observed in these two forest types during the last 7000 years, respectively (Fig. 2).

Second, the importance of vegetation was highlighted in our analyses of the relationships between biomass burning and the conifer *vs* broadleaf trees ratio (Table 1 and Fig. 5): biomass burning tended to be significantly higher during periods characterized by a higher proportion of conifers in landscapes, as was recently suggested by Girardin *et al.*^10^, Kelly *et al.*^23^, Brown & Giesecke^24^. Tree biomass was also positively correlated with variability of biomass burning (Table 1). But this correlation only holds in the set of analyses involving CCSM3 simulation runs; the conclusion that may be drawn from this result must therefore be considered with caution. From 7000 BP to present, biomass was largely dominated by coniferous species in the open coniferous forest/tundra, notably *Picea* (Supplementary Fig. 2). The broadleaf trees’ biomass remained low most of the time, resulting in a conifer *vs* broadleaf trees ratio above the long-term average since 5000 BP (>20, Fig. 2 and Supplementary Fig. 3). As for the dense boreal coniferous forest, conifers have dominated (*Picea*, Supplementary Fig. 2) since 7000 BP but the broadleaf tree component was higher with up to 10 t.ha-1 from 7000 to 6000 BP. Hence, low conifer biomass and conifer *vs* broadleaf trees ratio prevailed in the open coniferous forest/tundra and closed boreal coniferous forest *c.* 7000–5000 BP, consistent with lower biomass burning therein at that same period (Fig. 2).

Patterns of changes in vegetation features were quite different in other forest types. From 3000 BP to present, a decrease in broadleaf tree biomass (*Acer* and *Betula* Supplementary Fig. 2) was recorded in the boreal mixedwood forest (Fig. 2), parallel to the increase in the conifer *vs* broadleaf trees ratio. This ratio increase, with values above the long-term average after 2000 BP, was also partly triggered by a slight increase in conifers (mainly *Picea*) that occurred since 3000–4000 BP (Supplementary Fig. 2). This change in the vegetation composition occurred in parallel with an important decline in simulated summer precipitation (HadCM3 and CCSM3) (Fig. 3). The overall increase in the conifer *vs* broadleaf trees ratio and decline in summer precipitation therein are significantly correlated with the high biomass burning seen since 4000 BP (Figs 2 and 5). In temperate forests, slightly higher conifer *vs* broadleaf trees ratios between 5000 and 4000 BP and after 3000 BP, and low summer precipitation from 4000 BP to today, also coincided with periods of above-average biomass burning (albeit not statistically significant). Finally, our result did not identify statistical relationships between patterns of biomass burning and human population density and temperature fluctuations at millennial time scales (Table 1).

## Discussion

Fire activity reconstructions in eastern North America are inherently weighted toward the boreal mixedwood forests that encompass the majority of paleoecological sites (Fig. 1, 28 sites)^26^,^27^. The gradual increase in fire activity recorded over time at the subcontinental scale in this study and in previous ones e.g.^26^,^27^ are therefore likely the result of processes occurring within these forests. Our regional analysis provides a better scheme of these processes, allowing us to decipher the drivers and the fire dynamics in regions where records are scarcer, such as in the open and dense coniferous forests. Indeed, by considering different regions, we were able to underline that a high spatial and temporal variability in wildfire activity exists in eastern North America within the same biome (e.g., dense-coniferous and mixed boreal forests). Our results attribute spatial and temporal variability in long-term regional trends in biomass burning to complex and variable interactions between climate, particularly summer precipitation, and vegetation features.

Our data support previous paleoecological investigations that pinpointed the imprint of climate on biomass burning over the last 7000 years in open and dense boreal coniferous forests^16^,^28^. The observed decrease in biomass burning during the last 5000 years is most parsimoniously explained by climate change that caused a decrease in fire danger conditions. The mid-Holocene (6000–4000 BP) was characterized by warm and dry climate conditions during the fire season, which was limited to the summer months in the open coniferous forest/tundra and the closed boreal coniferous forest (Fig. 3)^29^,^30^. In contrast, colder and moister conditions prevailed afterward, during the Neoglacial period^31^,^32^, as suggested by the HadCM3 and CCSM3 simulations of summer precipitation. Neoglacial climate conditions were therefore less suitable to fire activity than mid-Holocene ones. The finding of diminishing fire activity from the mid-Holocene to the late-Holocene in northern forests agrees with other studies involving forest stand replacing fire histories^33^. Nevertheless in these regions the dynamics of fire activity cannot be attributed solely to modifications in climate: they may also have involved vegetation changes, as was suggested by the correlation analysis with the conifer *vs* broadleaf trees ratio. This relationship likely involves effects from gradual increases in the proportions of conifers and biomass burning from the mid-Holocene (Fig. 2). One might interpret this to indicate a post-glacial thickening of forests and consequent fuel loading under climate warming, both of which have contributed to sustaining greater fire activity. Since biomass burning followed the conifer *vs* broadleaf trees ratio, we can assume that biomass burning at that time was primarily controlled by fuel availability, a process that is also known to occur in high-altitude ecosystems^34^.

In boreal mixedwood forests, recent biomass burning is seemingly related to the proportion of flammable species in the ecosystem. In this region, summer cooling at ca. 4000 BP likely slowed down the activity of microorganisms and organic matter decomposition, particularly of conifer needles, a process that can contribute to soil acidification in our studied regions^35^,^36^. This soil modification is particularly suitable for the development of coniferous species such as *Picea mariana*. It is this increase in the conifer *vs* broadleaf trees ratio *c.* 4000 BP that contributed to maintaining high fire activity under circumstances of unfavourable climate conditions for fire (e.g. colder and overall moister; Fig. 3). The abundant presence of broadleaf trees in boreal mixedwood landscapes prior to 4000 BP may imply that fires mainly occurred during the spring before the broadleaf trees leaf out, when the light reaching the ground dries fuels. These conditions are less easily achieved during summer when the broadleaf tree canopy captures moisture in the understory^37^,^38^. After *c.* 4000 BP, more abundant coniferous and therefore flammable components extended fire-prone conditions over the whole fire season (April to September). This hypothesis is valid if we assume that the coniferous and broadleaf trees are interspersed at a fine spatial scale, which cannot be confirmed by the pollen analysis alone, or if continuous patches of conifers burn frequently in a landscape that has a larger broadleaf component. Whatever the landscape structure, the additional lengthening of the fire-prone season, associated with the above-mentioned climate and vegetation processes, likely favoured sustained fire activity during the late Holocene.

In the temperate forest, the most important change in biomass burning was recorded during the last 200 years, with a sharp increase 1650–1750 AD towards levels comparable to the mid-Holocene maxima (Fig. 2; window-time 200 years). The increase in biomass burning was uncoupled with vegetation features and there is no indication of an increase in fire weather hazards in these regions during the 20th century^39^. It is possible that this rise in fire activity may be partly human induced. But it is not an easy task to record significant correlation between human activities and biomass burning pattern, because it is hard to model past population density dynamics. Moreover, one cannot totally rule out the influence of native populations on fire activity before European colonization, and more research on this topic is clearly advisable^40^. The role of native populations in fire activity must be fully considered in western parts of North America and in the Prairies but, in regard to known Aboriginal cultural practices, it is less so in eastern forests. After a large decrease in native North American populations from disease by 1600 AD as a result of the initial European colonization^41^, it is admitted that the rise in European populations would have affected the fire regime particularly in the temperate forest area during the 1800’s AD^26^,^42^. After 1800 AD the first European farmers influenced the fire regime through slash and burn practices to clear land for agriculture or to control pests. The mutation of societies from first farmers to extended industrialization that is accompanied elsewhere in the Americas by a decline in biomass burning^13^,^27^ is not observed here in the temperate forest. For example, in the Prairies, tillage and fire suppression west of the Great Lakes resulted in a decrease in fire activity which seems noticeable in the biomass burning *c.* 1900 AD^43^. Conversely, in eastern forests, burning for land clearance, heating, and industry continued during the 19th and 20th centuries, which could explain the observed increase in biomass burning in the temperate forest (Fig. 2)^43^.

Climate projections for the end of the 21st century indicate a rise in temperatures with continuing increases in atmospheric greenhouse gas concentrations^44^. There is now a wealth of indications that this warming will cause an increase in fire activity in global forests^45^. Our statistical analyses failed to detect past influences of temperatures on biomass burning but this should not be taken as an example for the future. The comparison of the CCSM3 and HadCM3 model simulations of temperatures brings to our attention that past changes in this variable over our studied regions are still uncertain as there is a disagreement between model simulations inducing large uncertainties (Fig. 3). Climate model uncertainty inevitably influences our capacity to decipher the drivers and the dynamics of fire in the past and in the future. This uncertainty adds up to processes that are currently not fully understood and may include permafrost degradation, shifts in vegetation community or impacts from major changes in land use^46^. There is a high probability that current temperatures are close to levels experienced during the mid-Holocene and the probability of seeing future temperatures exceed these levels increases as we move further into the 21st century^47^. The magnitude of these changes is important to the point that future fire weather hazards will rise, regardless of precipitation trajectories particularly in the boreal biome^48^. Nevertheless, as shown in this work, past biomass burning varied in parallel with changes in vegetation composition, an indication that biomass burning is also fuel-mediated. Modifications in landscape structure from human activities, coupled with vegetation shifts or recurrent fires over short intervals, could modify fuel availability and feedback negatively on fire ignition and spread^13^,^49^,^50^.

## Methods

### Charcoal sites selection

We used 52 charcoal records located in eastern North America from the Global Charcoal Database version 3 and 12 additional published records not yet included in the database from Ali *et al.*^16^ and Oris *et al.*^30^, for a total of 64 charcoal records (Fig. 1). Sites were classified into the four vegetation zones according to potential vegetation maps produced by Ramankutty & Foley^51^ and the classification used by Girardin *et al.*^10^. Ramankutty & Foley^51^ used a single potential vegetation class, evergreen/deciduous mixed forest/woodland, to describe both open coniferous woodlands located above the closed boreal coniferous forest in the north and the boreal mixedwood forests located to the south. To correct for this bias, we assigned charcoal sites located north of the closed boreal coniferous forest belt to the open coniferous forest/tundra class and the sites south of it were assigned to the boreal mixedwood forest.

### Charcoal records of biomass burning

We developed fire histories in regard to vegetation zones in order to evaluate the spatial variability of processes, notably controlled by vegetation dynamics. The broad patterns of vegetation are the result of long-term ecological processes, including post-glacial migrations, competition, soil formation, etc. that occurred after deglaciation deglaciation. Considering the vegetation gradient present in this study, and taking into account that deglaciation was not synchronous across our transect, we only focused on the last 7000 years. Moreover during the last 6000 years, the area was totally deglaciated^52^ and vegetation zones could be assumed to be spatially stable^53^.

Charcoal values were converted to accumulation rates (CHAR) by multiplying charcoal concentrations with sediment accumulation rates assessed using records age-depth models. Because of large variations between CHAR among and within the different records, which occur as a result of analytical and methodological differences or differences related to site characteristics, a standardization procedure was required. We used the transformation procedure proposed by Power *et al.*^2^ that includes a minmax rescaling of CHAR values followed by a Box-Cox transformation to homogenize the within-records variance, and finally a Z-score transformation. Transformed charcoal records of each vegetation zone were then bootstrap resampled 999 times with a moving window procedure using non-overlapping bins of 10 years. Resampled series were then smoothed using a locally weighted scatterplot smoother using a window half width of 500 years. Regional charcoal composite series mean (biomass burning) and 90% confidence intervals were calculated by averaging the smoothed and bootstrapped series^54^. A smoother with a 200-year bandwidth was calculated in order to highlight high-frequency trends in the transformed charcoal series.

### Vegetation biomass

Although biomass reconstructions using transfer functions have been occasionally performed, large-scale studies were generally lacking due to the difficulties to obtain quantitative and qualitative data on modern vegetation to develop functions with a high predictive power linking bio-proxy assemblages to biomass. The availability of high-resolution modern biomass data over large climatic and vegetation gradients^22^ enabled us to produce robust transfer functions for the main eastern North American tree genera (Supplementary Fig. 4).

We used the Modern Analogue Technique (MAT) transfer function applied to pollen records in order to reconstruct the past tree biomass for the main tree genera in eastern North America. The modern pollen database we used was the North American Surface Sample Dataset by Whitmore *et al.*^55^. From this dataset we extracted Canadian modern samples located east of 100 °W and estimated the biomass only for eastern main tree genera i.e. *Abies*, *Acer*, *Betula*, *Picea*, *Pinus* and *Populus*. In this analysis we only considered the genus or family level depending on pollen taxa, in order to down-weight pollen identification bias. In order to establish a correspondence between modern pollen assemblages and tree biomass, we used vegetation maps produced by Beaudoin *et al.*^22^. These maps consisted of tree biomass estimates at 250 × 250 m across Canada derived from classification algorithms applied to satellite images. Prior to MAT, we estimated the optimal distance for compiling biomass for each taxa by calculating the predictive R-squared value between tree biomass and modern pollen percentages for increasing distances such as radius= 1, 5, 15,…, 150 km. The distances that maximized the predictive R-squared were 30 km for *Abies*, 15 km for *Acer*, 15 km for *Betula*, 20 km for *Picea*, 120 km for *Pinus*, and 15 km for *Populus*. These distances are consistent with empirical studies of pollen source-area^56^ and models of pollen dispersal^57^. We used these distances for compiling the average biomass for each tree genus around each modern pollen sample. We used these biomass values for reconstructing MAT transfer functions and evaluated their performance by looking at predicted *vs* observed biomass distribution, associated correlation coefficients and Root-Mean-Square Error of Prediction (RMSEP, Supplementary Fig. 4). The number of analogues needed to reconstruct past biomass (k) for each genus was evaluated by bootstrap re-sampling the modern dataset split into training and test sets^58^,^59^. The MAT transfer functions were then applied to fossil pollen assemblages extracted from the Neotoma database that were primarily aggregated to the same taxonomic levels we used for the modern dataset (see Table A2 for the full citation list of the Neotoma sites). Age-depth models in the Neotoma database were corrected according to Blois *et al.*^60^. Fossil pollen sites were selected according to their location, i.e. we only included sites located less than 100 km apart from a charcoal site. In order to evaluate the temporal trends in tree biomass, we used the same procedure as biomass burning that involved: (i) resampling tree biomass within 10 years overlapping windows, (ii) resampling these series using a bootstrap procedure 999 times, (iii) smoothing the resampled series using a locally weighted scatterplot smoother with a window half width of 500 years (a 200-year scatterplot smoother was also calculated to highlight the short-term trends in the data), and (iv) calculating composite mean and 90% confidence intervals from the smoothed and bootstrapped series.

### Human population density

Population density estimates and land use changes were obtained from the History Database of the Global Environment (HYDE 3.1) database covering the period 12,000 BP to present^19^. HYDE population data consisted of the total population count for 5′ resolution pixels located worldwide. For each date available in the HYDE database, we summed the population for all pixels located in a radius of 100 km around each lake (corresponding to an area of *c.* 31,400 km^2^). Then we calculated the average population density (i.e. pop count/area) around charcoal sites for each vegetation zone during the last 7000 years.

### Climate reconstructions

We used paleoclimatic simulations provided by the UK Universities Global Atmospheric Modeling Program to develop a mechanistic understanding of the climatic variations associated with the reconstructed paleofire frequency. These simulations were performed with the Hadley Centre climate model HadCM3^61^, which is a state-of-the-art global climate model (GCM) used in both the third and fourth assessment reports of the Intergovernmental Panel on Climate Change (2001, 2007). Additionally we used paleoclimatic simulations from the Community Climate System Model^62^, CCSM3. These GCMs are a three-dimensional time-dependent numerical representation of the atmosphere, oceans and sea ice and their phenomena over the entire Earth, using the equations of motion and including radiation, photochemistry, and the transfer of heat and water vapour. The GCM simulations used in the present study consist of climatic averages at 1000-year intervals (i.e. maximum temporal resolution available) covering the last 21,000 and 22,000 years at a spatial resolution of 2.5° in latitude by 3.75° in longitude and 1.4° in latitude and longitude for HadCM3 and CCSM3 respectively. For each millennium interval, anomalies for air temperature (the difference between a given millennium and the pre-industrial (AD c. 1750) period) and precipitation (the percentage of change between a given millennium and the pre-industrial period) were computed. A downscaling method was used in which means of HadCM3 GCM anomalies of temperature and precipitation were applied to Climate Research Unit spatial grids TS 3.1 period AD 1901–2008^63^. For the downscaling of CCSM3 GCM the reader is referred to Veloz *et al.*^64^. Downscaled temperatures and precipitation were then extracted at a radius of 100 km around charcoal sites in order to achieve high spatial resolution comparisons. Finally, we calculated seasonal anomalies by averaging spring (March, April, May) and summer (June, July, August) monthly temperatures and precipitation, for the four vegetation zones.

### Statistical analyses

We explored the relationships between variability in vegetation, climate, and population densities, and multi-millennial variability in regional biomass burning activity *via* the semipartial Spearman rank correlation (sr)^65^. Semipartial correlation is the correlation of two variables with variation from other variables removed only from the second variable. The hypothesis *“variability in aboveground tree biomass is significantly correlated with multi-millennial variability in biomass burning”* was tested after removing the effects on biomass burning of the spring and summer temperature and precipitation (obtained from the HadCM3 climate model), conifer *vs* broadleaf trees ratio, and population densities. The analysis was done on all four regions combined after downsampling biomass burning, vegetation, and human population density data to the resolution of the climate records (i.e., 1000 years), and normalizing data (zero mean) by region (total sample size N = 4 regions * 8 millennia= 32). The semipartial correlation analysis was redone after substituting the HadCM3 data with CCSM3 data. The null hypothesis of no relationship between aboveground tree biomass and regional biomass burning from 7000 BP to present was to be rejected when p < 0.05. The semipartial correlation test was done iteratively after substituting the dependent variable with another one until all variables were tested for their unique contribution. The significant explaining variables brought about by the correlation analysis were further analyzed using partial least squares regression in order to explore the functional form of the relationship between regional biomass burning and the climatic or vegetation forcing factors.

### Data sources

The North American Surface Sample Dataset^55^ was obtained from http://www.geography.wisc.edu/faculty/williams/lab/Downloads.html. Fossil pollen sites were obtained from the Neotoma database http://www.neotomadb.org. Actual vegetation biomass rasters^22^ were obtained from Canada’s National Forest Inventory Service https://nfi.nfis.org. Human population data were obtained from the HYDE 3.1 database^19^ available at http://themasites.pbl.nl/tridion/en/themasites/hyde/. HadCM3 paleoclimatic simulations were obtained from http://www.bridge.bris.ac.uk/resources/simulations; CCSM3 paleoclimatic simulations were obtained from http://ccr.aos.wisc.edu/resources/data_scripts/.

Charcoal data were obtained from the Global Charcoal Database available at http://gpwg.org. All calculations were done using the R programming language and relied on the use of the paleofire package^66^ for charcoal and tree biomass syntheses, the analogue package^58^ for the modern analogue technique, the ppcor package for semipartial correlations and the pls package for partial least squares regressions.

## Additional Information

**How to cite this article**: Blarquez, O. *et al.* Regional paleofire regimes affected by non-uniform climate, vegetation and human drivers. *Sci. Rep.* **5**, 13356; doi: 10.1038/srep13356 (2015).

## Supplementary Material

Supplementary Information

Supplementary Table 1

Supplementary Table 2

## Figures and Tables

**Table 1:** Results of the semipartial Spearman rank correlation analyses of the regional biomass burning history with the vegetation, climate and human population density features on millennial time scales.

Variable under analysis	Climate models			
	HadCM3		CCSM3	
	sr	p-value	sr	p-value
Conifer *vs* broadleaf trees ratio	0.54	0.002	0.56	0.001
Aboveground tree biomass	0.21	0.286	0.45	0.013
Population density	−0.06	0.778	0.01	0.947
Spring temperature	−0.05	0.799	0.3	0.126
Summer temperature	0.14	0.473	−0.15	0.449
Spring precipitation	0.04	0.83	0.24	0.223
Summer precipitation	−0.37	0.049	−0.38	0.041

Significant correlation coefficients (sr) and *p*-values are underlined.
