# Supplementary material for: Regional paleofire regimes affected by non-uniform climate, vegetation and human drivers
Source: Sci Rep. 2015 Sep 2;5:13356. doi: 10.1038/srep13356 (PMC4557068; doi:10.1038/srep13356)
Supplement: Supplementary Information [file srep13356-s1.pdf]

Regional paleofire regimes affected by non-uniform climate, vegetation and human drivers

Blarquez Olivier[1,*,+], **Ali Adam A.**[2,+], **Girardin Martin P.**[3,+], **Grondin Pierre**[4], **Fréchette Bianca**[5], **Bergeron Yves**[6,7], **and Hély Christelle**[2]

[1]Département de Géographie, Université de Montréal, Montréal, Québec, Canada
[2]Institut des Sciences de l'Evolution de Montpellier, CNRS - IRD - Université Montpellier 2 - EPHE, Montpellier, France
[3]Natural Resources Canada, Canadian Forest Service, Laurentian Forestry Centre, Quebec, QC, Canada
[4]Ministère des Forêts, de la Faune et des Parcs, Direction de la recherche forestière, Québec, Canada
[5]Centre de recherche en géochimie et géodynamique, Université du Québec à Montréal, Montréal, Québec, Canada
[6]Centre d'étude de la Forêt, Université du Québec à Montréal, Montréal, Québec, Canada
[7]Natural Sciences and Engineering Research Council of Canada Industrial Chair in Sustainable Forest Management, Forest Research Institute, Université du Québec en Abitibi-Témiscamingue, Rouyn-Noranda, Québec, Canada
[*]corresponding author: blarquez@gmail.com
[+]these authors contributed equally to this work

ABSTRACT

Climate, vegetation and humans act on biomass burning at different spatial and temporal scales. In this study, we used a dense network of sedimentary charcoal records from eastern Canada to reconstruct regional biomass burning history over the last 7000 years at the scale of four potential vegetation types: open coniferous forest/tundra, boreal coniferous forest, boreal mixedwood forest and temperate forest. The biomass burning trajectories were compared with regional climate trends reconstructed from general circulation models, tree biomass reconstructed from pollen series, and human population densities. We found that non-uniform climate, vegetation and human drivers acted on regional biomass burning history. In the open coniferous forest/tundra and dense coniferous forest, the regional biomass burning was primarily shaped by gradual establishment of less climate-conducive burning conditions since 5000 years. In the mixed boreal forest an increasing relative proportion of flammable conifers in landscapes since 2000 BP contributed to maintaining biomass burning constant despite climatic conditions less favourable to fires. In the temperate forest, biomass burning was uncoupled with climatic conditions and the main driver was seemingly vegetation until European colonisation, i.e. 300 BP. Tree biomass and thus fuel accumulation modulated fire activity, an indication that biomass burning is fuel-dependent and notably upon long-term co-dominance shifts between conifers and broadleaved trees.

Supplementary information

Table S1 (xls format): Citation list for the charcoal sites used to reconstruct the past regional biomass burning.

Table S2 (xls format): Citation list for the Neotoma pollen sites used to reconstruct the past regional biomass per tree genus.

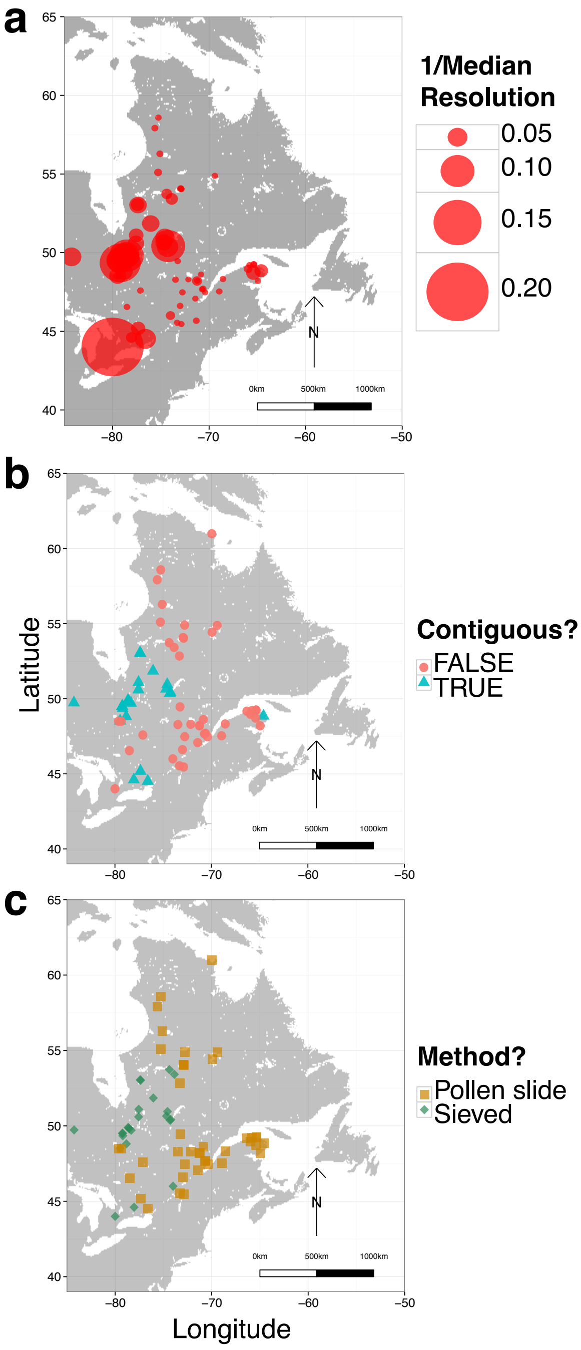

Figure 1. Summary maps of the main feature of the charcoal sites: (a) inverse of the median resolution of charcoal records, (b) contiguous (i.e. sites sampled contiguously) and non contiguous charcoal records and (c) charcoal records obtained from pollen slides analysis or from sieved charcoals. The maps have been produced using R.

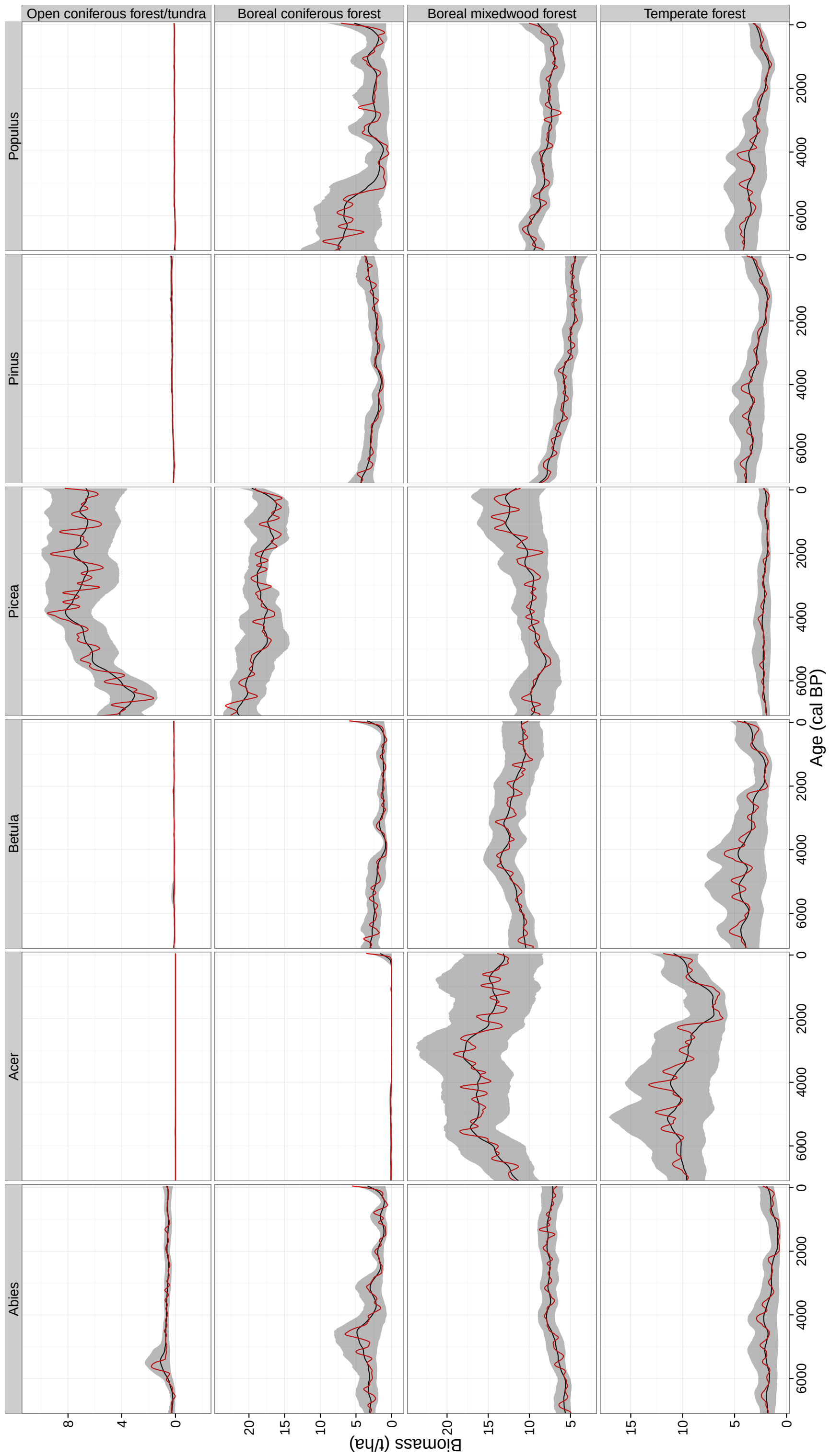

Figure 2. Predicted total aboveground biomass for the main tree genera in eastern North America and for the four regions. The black and red lines corresponds to the scatter plot smoother calculated using a 500 and 200 year window half width, respectively. The grey area represents the 95% confidence interval calulated using the bootstrap procedure.

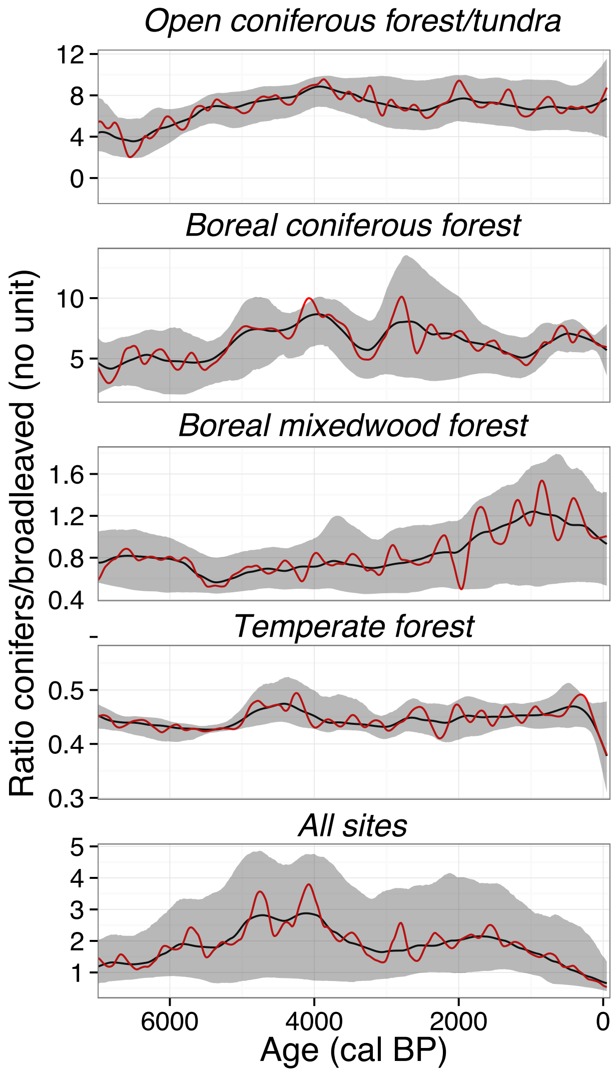

Figure 3. conifer *vs* broadleaf trees ratio. The black and red lines correspond to the scatter plot smoother calculated using a 500 and 200 year window half width, respectively. The grey area represents the 95% confidence interval calculated using the bootstrap procedure (calculated on the 500-yr trend).

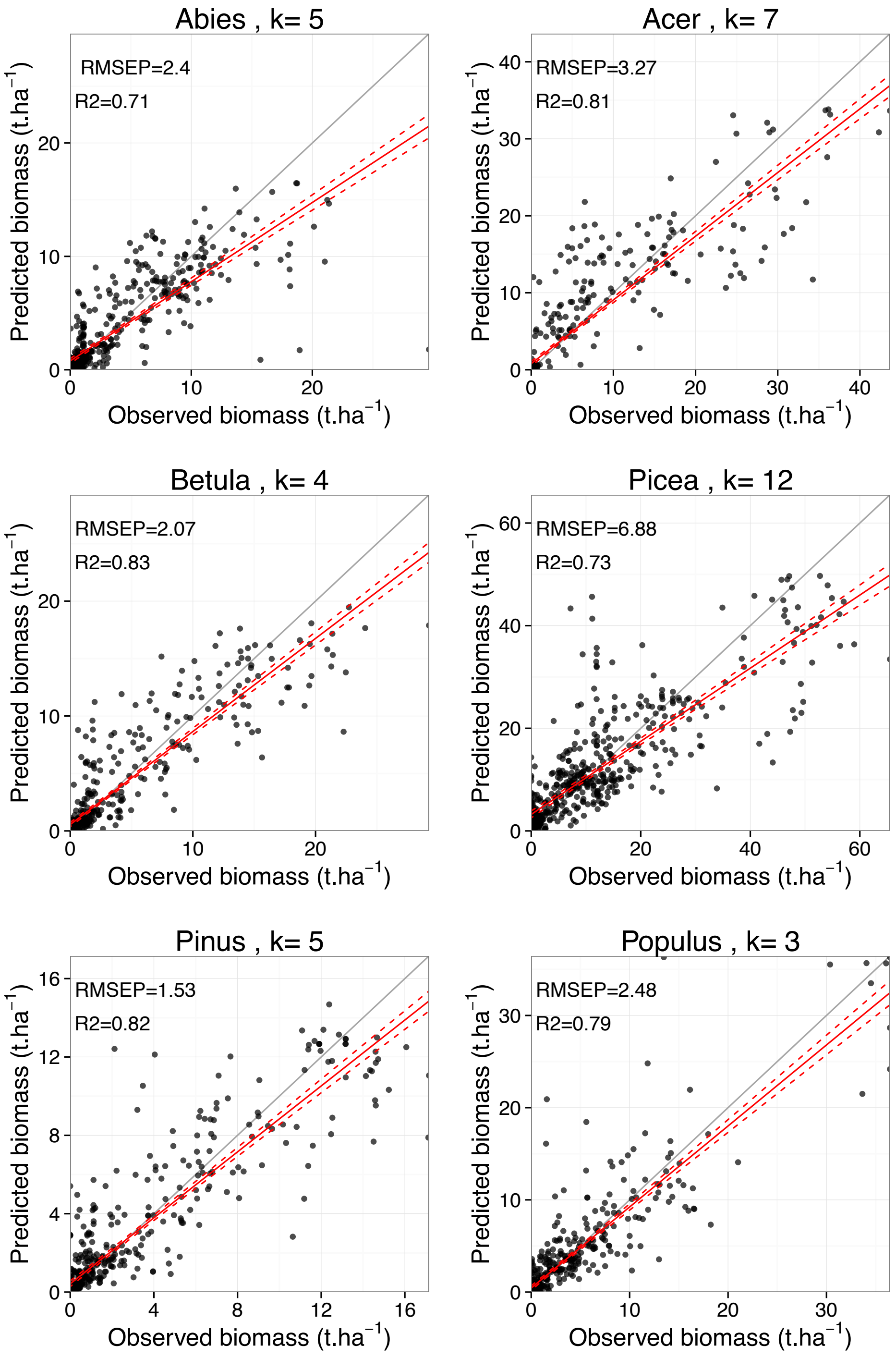

Figure 4. Observed *vs* predicted values for the Modern Analogue Technique. Root Mean Square Error of Prediction (RMSEP) and determination coefficients (R2) are indicated on each graph.
